# Supplementary figures and images for: Octopamine drives honeybee thermogenesis
Source: eLife. 2022 Mar 15;11:e74334. doi: 10.7554/eLife.74334 (PMC8923666; doi:10.7554/eLife.74334)

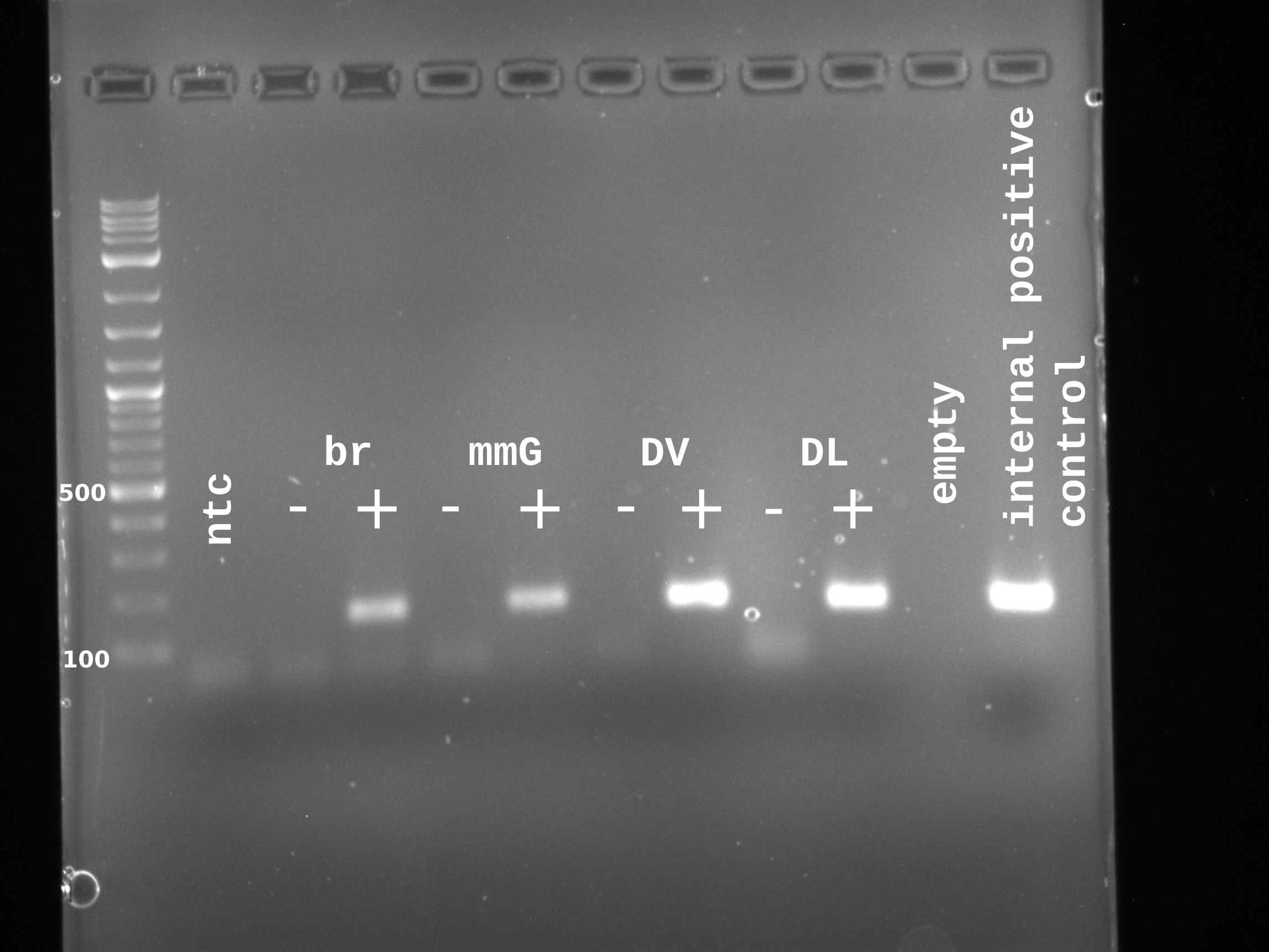

Supplement: Figure 3—source data 1. [file elife-74334-fig3-data1.zip › Figure_3_raw_gels_labelled/2020-10-30_12-43-55_AmOARalpha1_labelled.png]

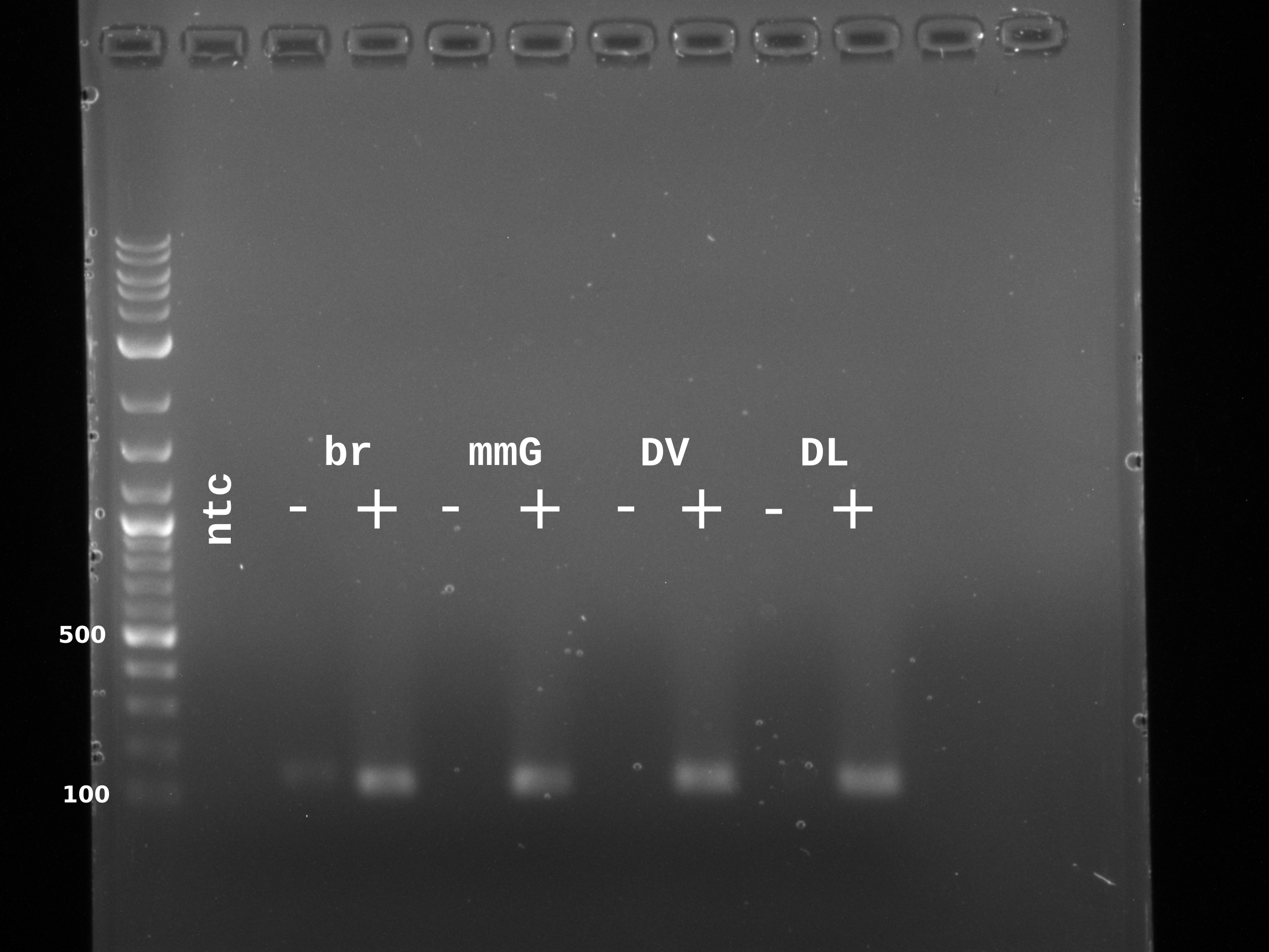

Supplement: Figure 3—source data 1. [file elife-74334-fig3-data1.zip › Figure_3_raw_gels_labelled/2020-11-02_12-45-58_AmGAPDH_labelled.png]

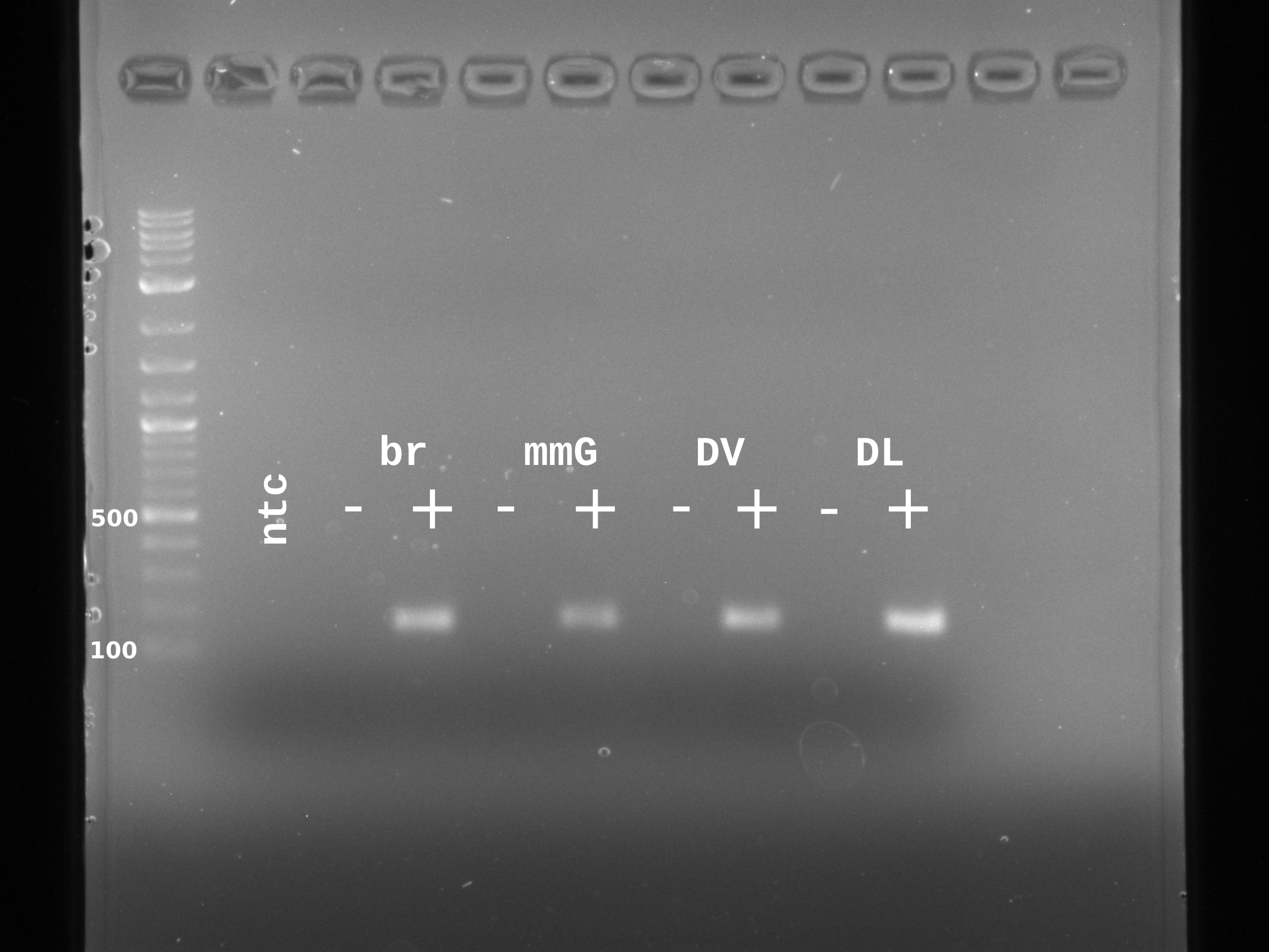

Supplement: Figure 3—source data 1. [file elife-74334-fig3-data1.zip › Figure_3_raw_gels_labelled/2020-11-03_09-07-36_AmOARbeta2_labelled.png]

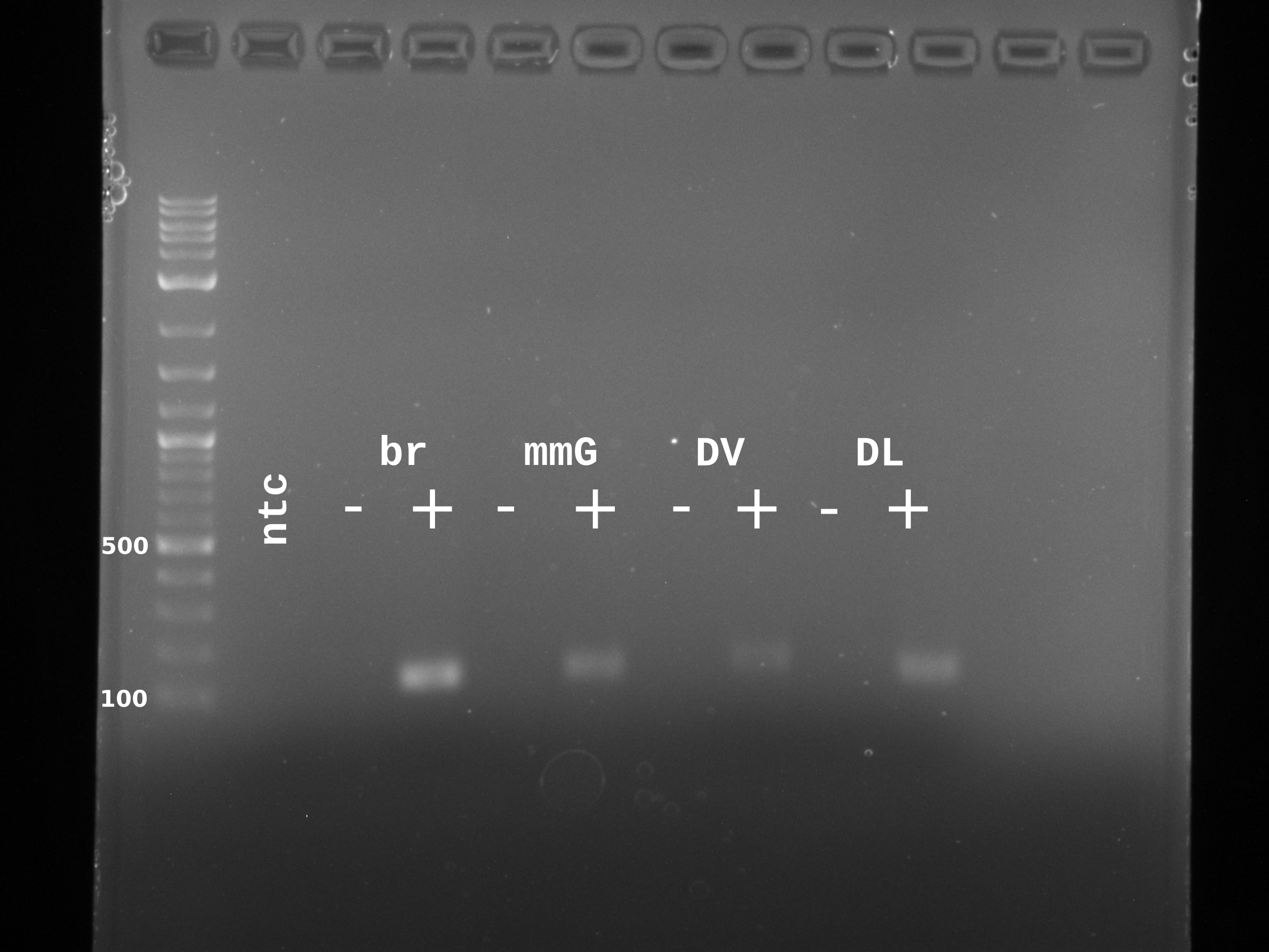

Supplement: Figure 3—source data 1. [file elife-74334-fig3-data1.zip › Figure_3_raw_gels_labelled/2020-11-03_09-03-03_AmOARbeta1_labelled.png]

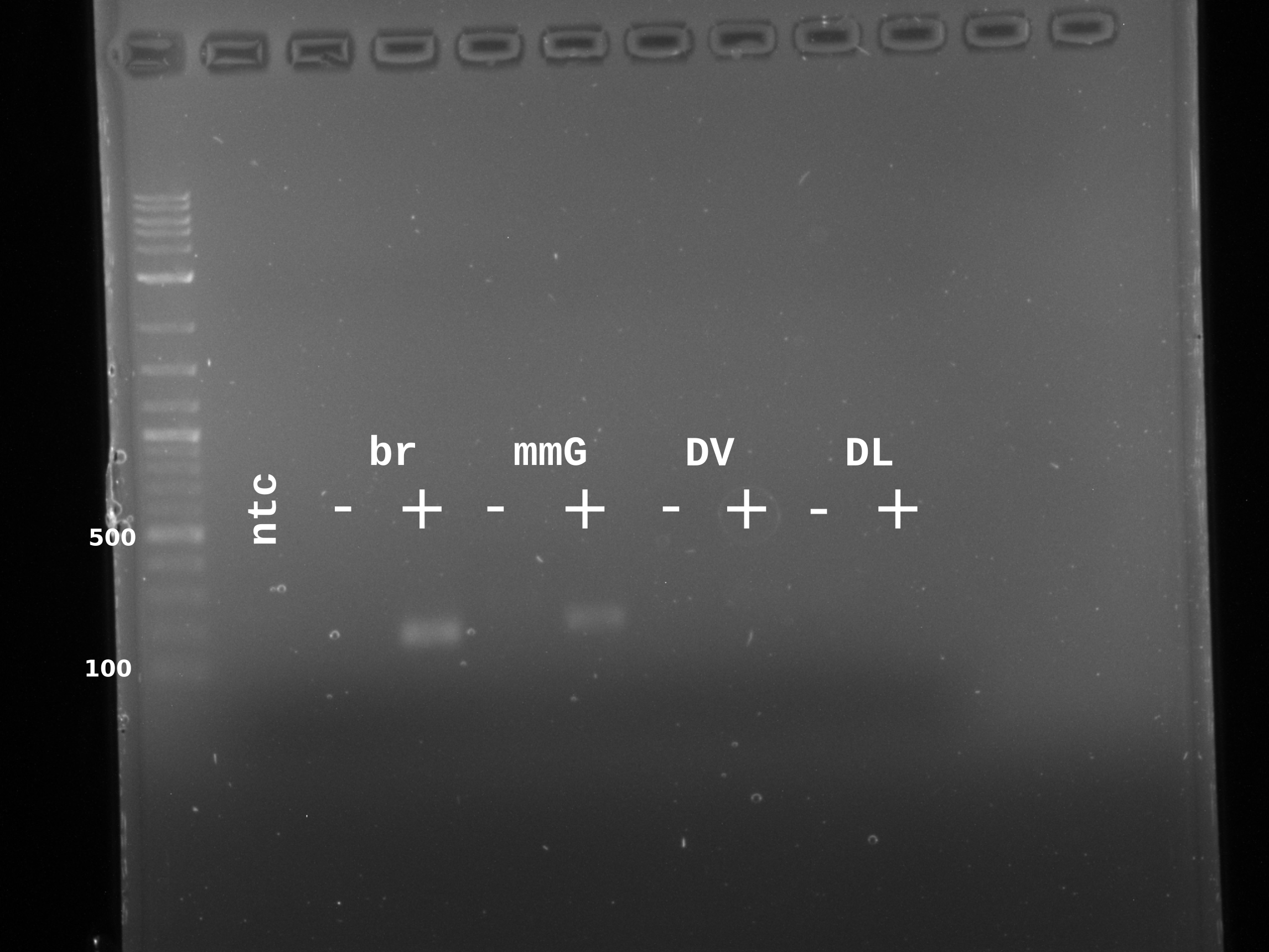

Supplement: Figure 3—source data 1. [file elife-74334-fig3-data1.zip › Figure_3_raw_gels_labelled/2020-11-03_10-18-19_AmTAR1_labelled.png]

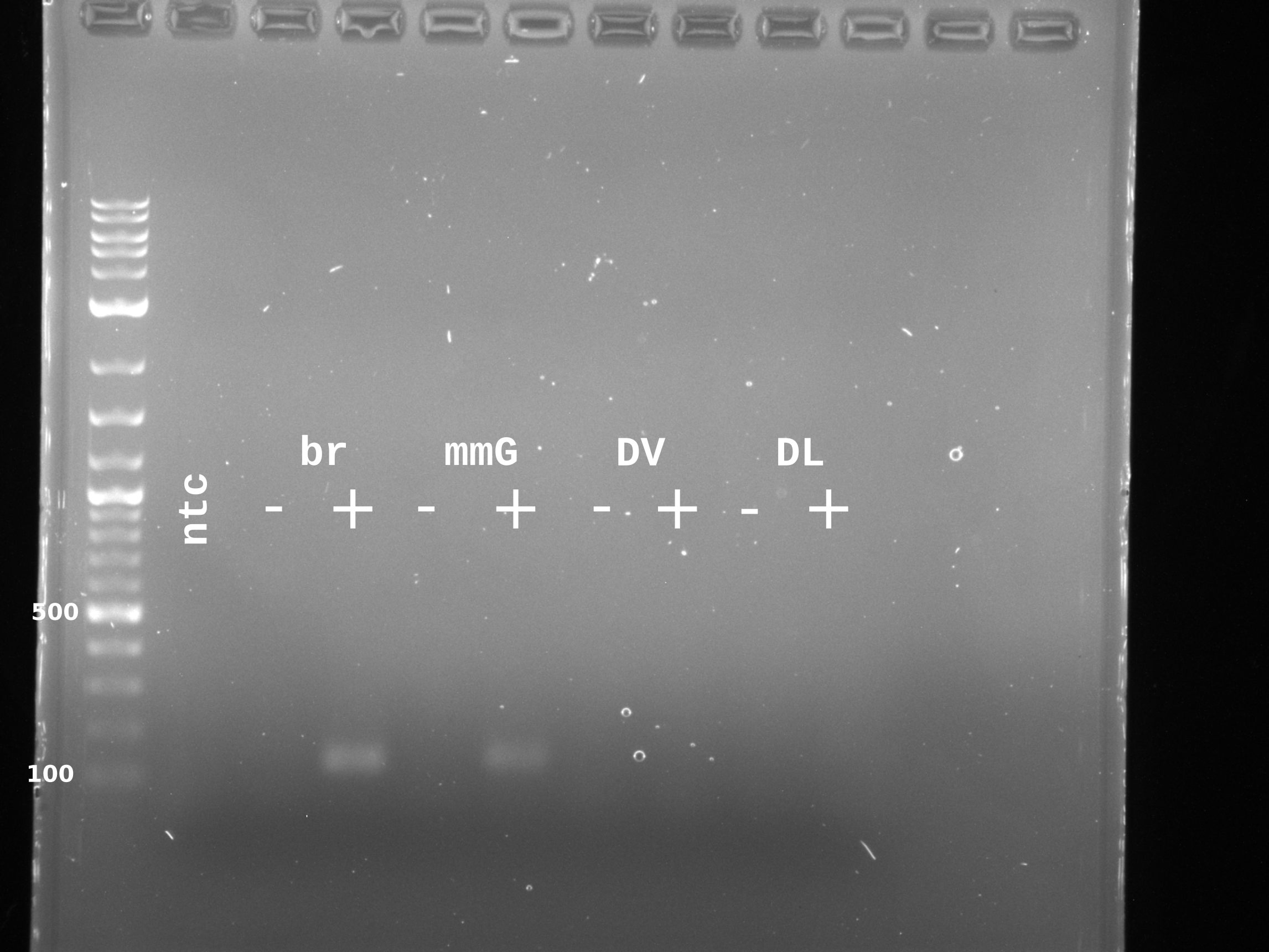

Supplement: Figure 3—source data 1. [file elife-74334-fig3-data1.zip › Figure_3_raw_gels_labelled/2020-11-02_12-45-58_AmTAR2_labelled.png]

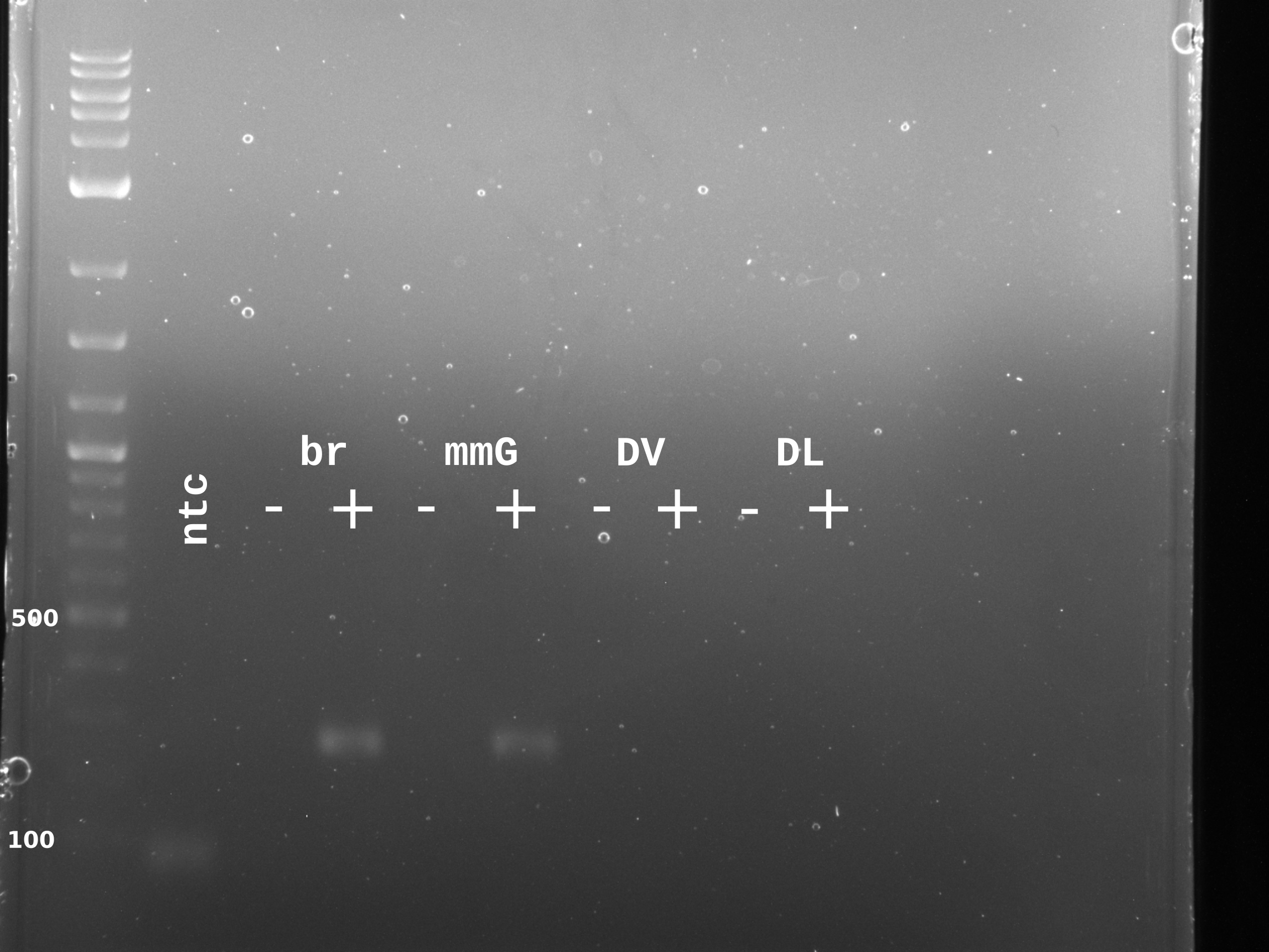

Supplement: Figure 3—source data 1. [file elife-74334-fig3-data1.zip › Figure_3_raw_gels_labelled/2020-11-05_10-52-27_AmOARalpha2_labelled.png]

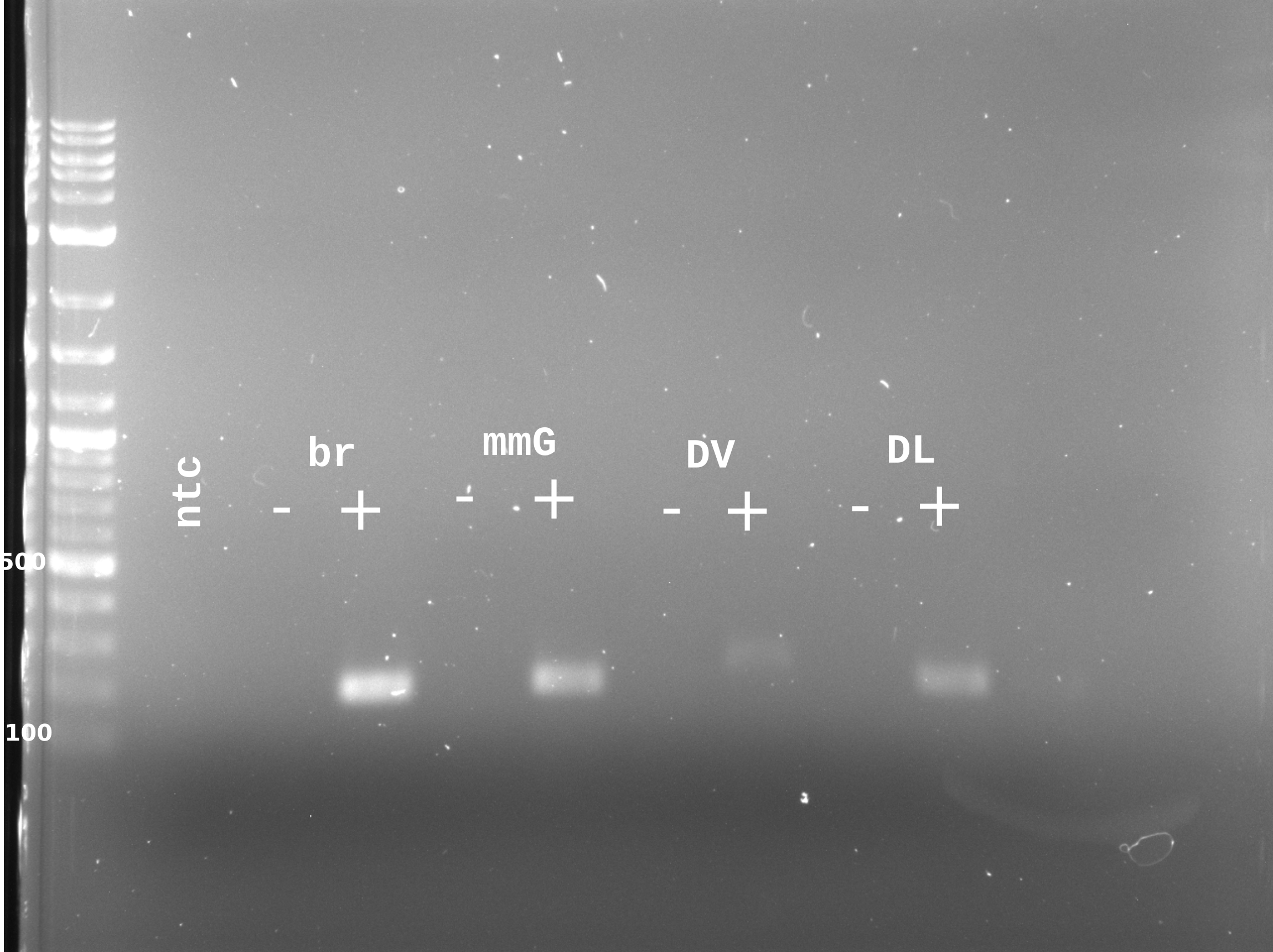

Supplement: Figure 3—source data 1. [file elife-74334-fig3-data1.zip › Figure_3_raw_gels_labelled/2020-11-05_09-30-20_AmOARbeta34_labelled.png]

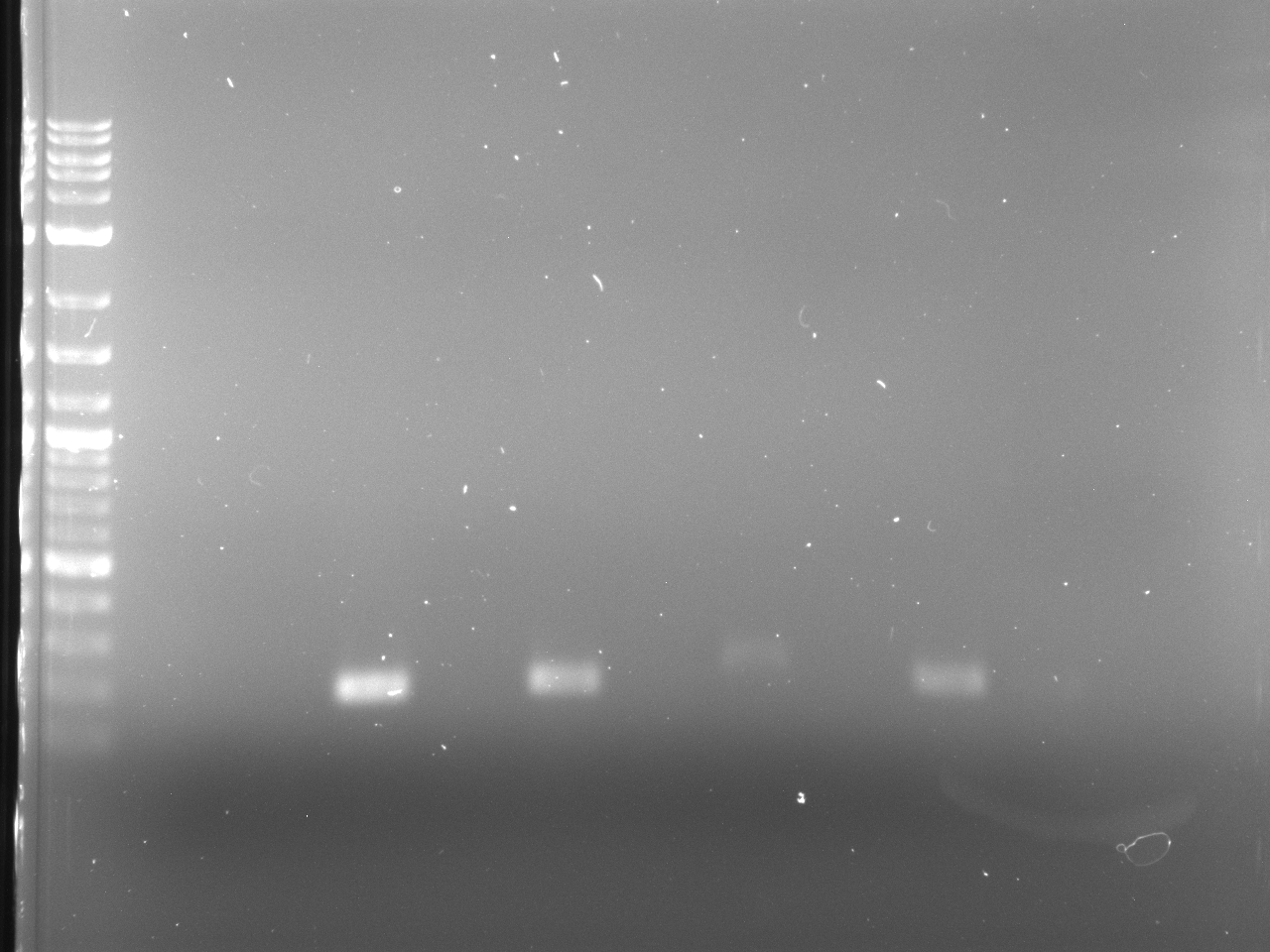

Supplement: Figure 3—source data 2. [file elife-74334-fig3-data2.zip › Figure_3_raw_gels_unedited/2020-11-05_09-30-20_AmOARbeta34.tif]

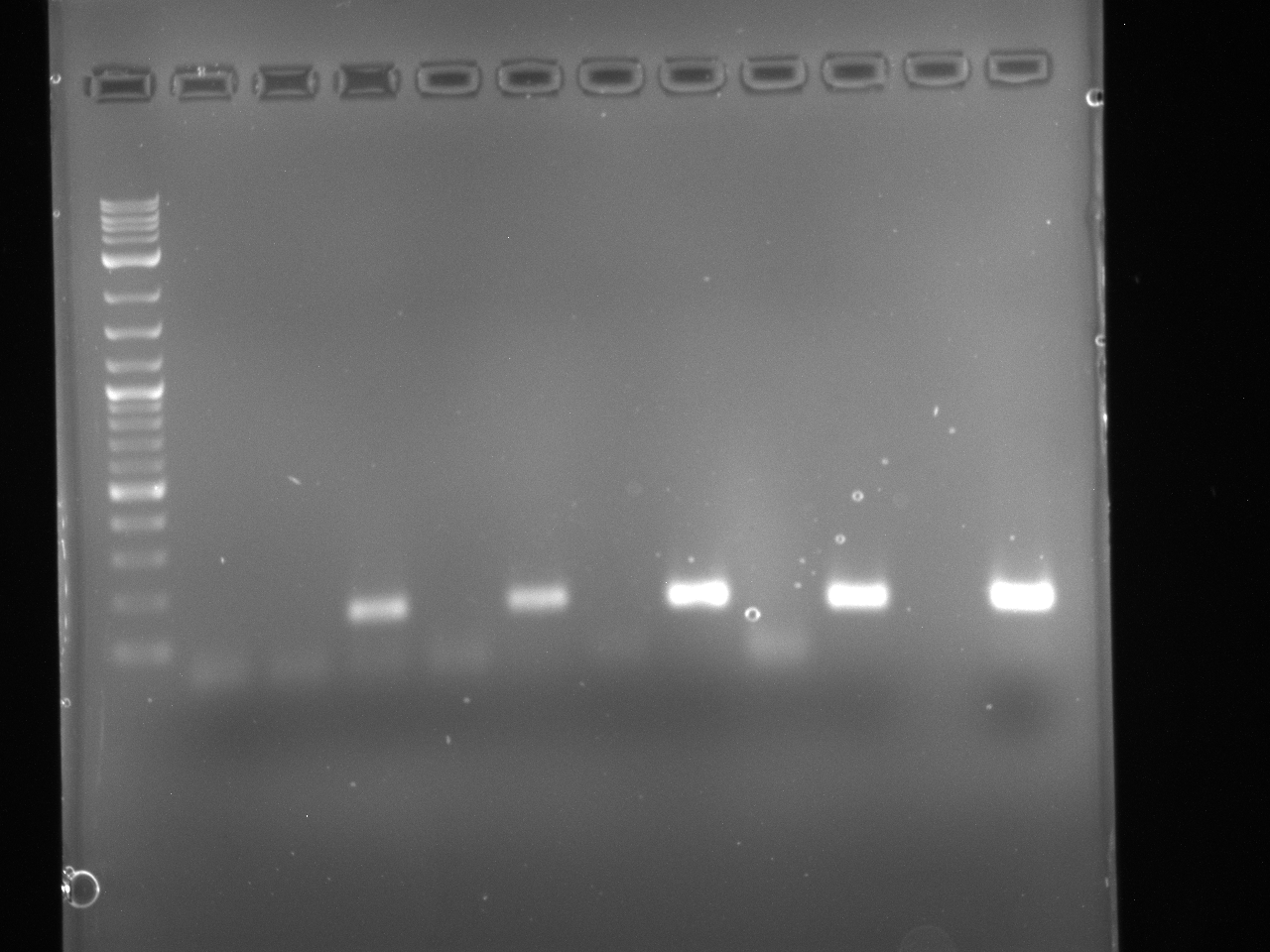

Supplement: Figure 3—source data 2. [file elife-74334-fig3-data2.zip › Figure_3_raw_gels_unedited/2020-10-30_12-43-55_AmOARalpha1.tif]

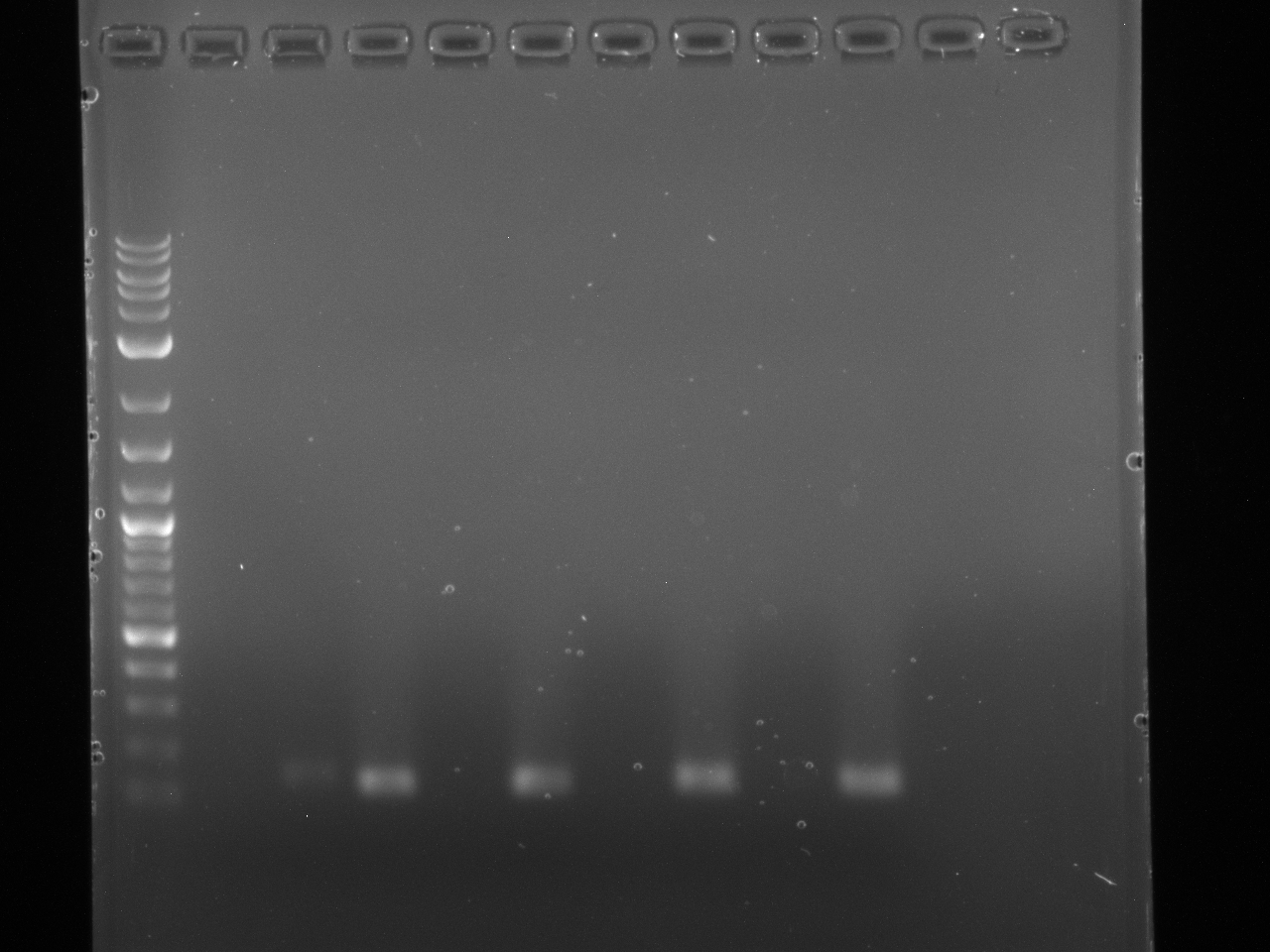

Supplement: Figure 3—source data 2. [file elife-74334-fig3-data2.zip › Figure_3_raw_gels_unedited/2020-11-02_12-45-58_AmGAPDH.tif]

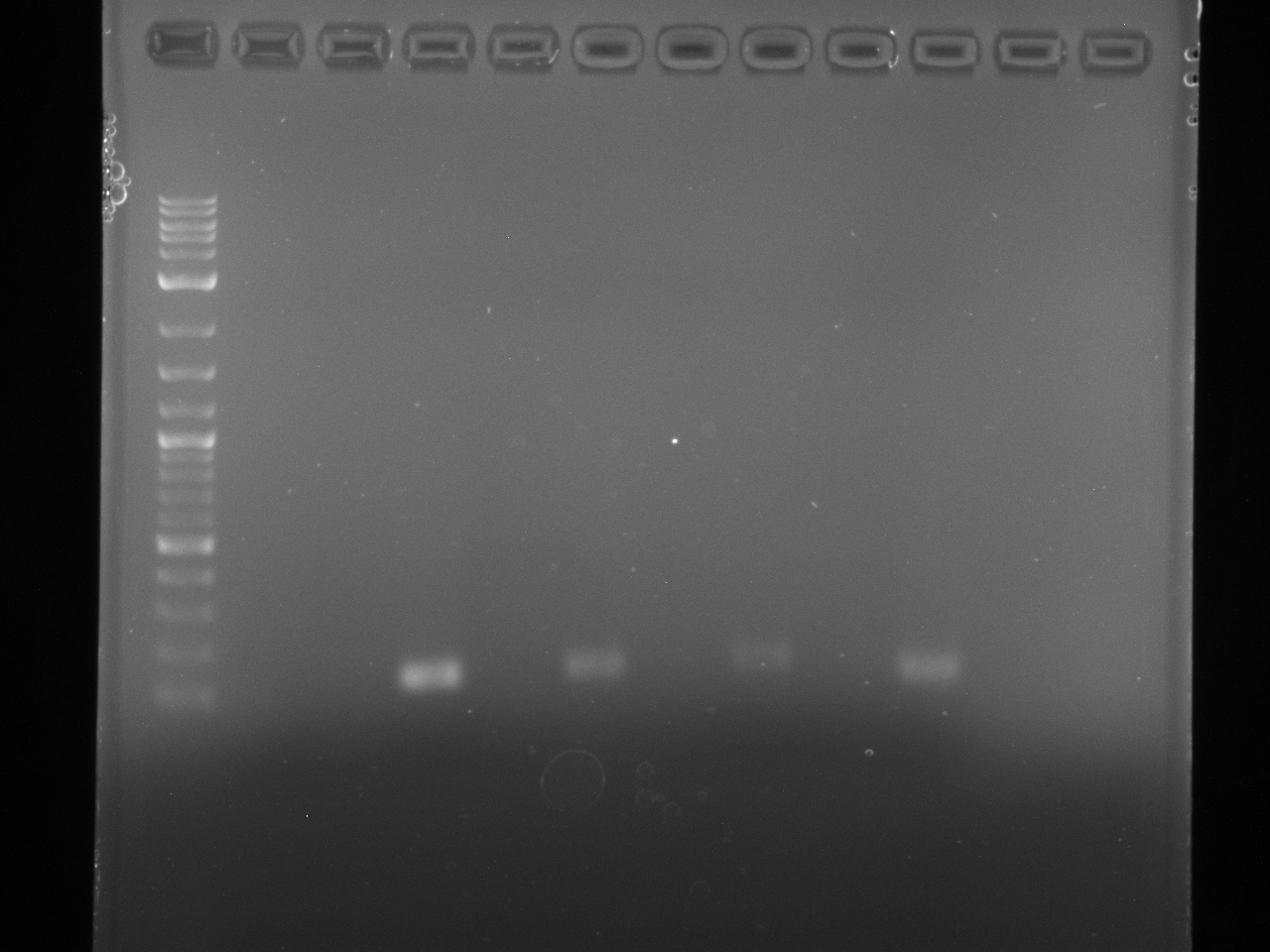

Supplement: Figure 3—source data 2. [file elife-74334-fig3-data2.zip › Figure_3_raw_gels_unedited/2020-11-03_09-03-03_AmOARbeta1.tif]

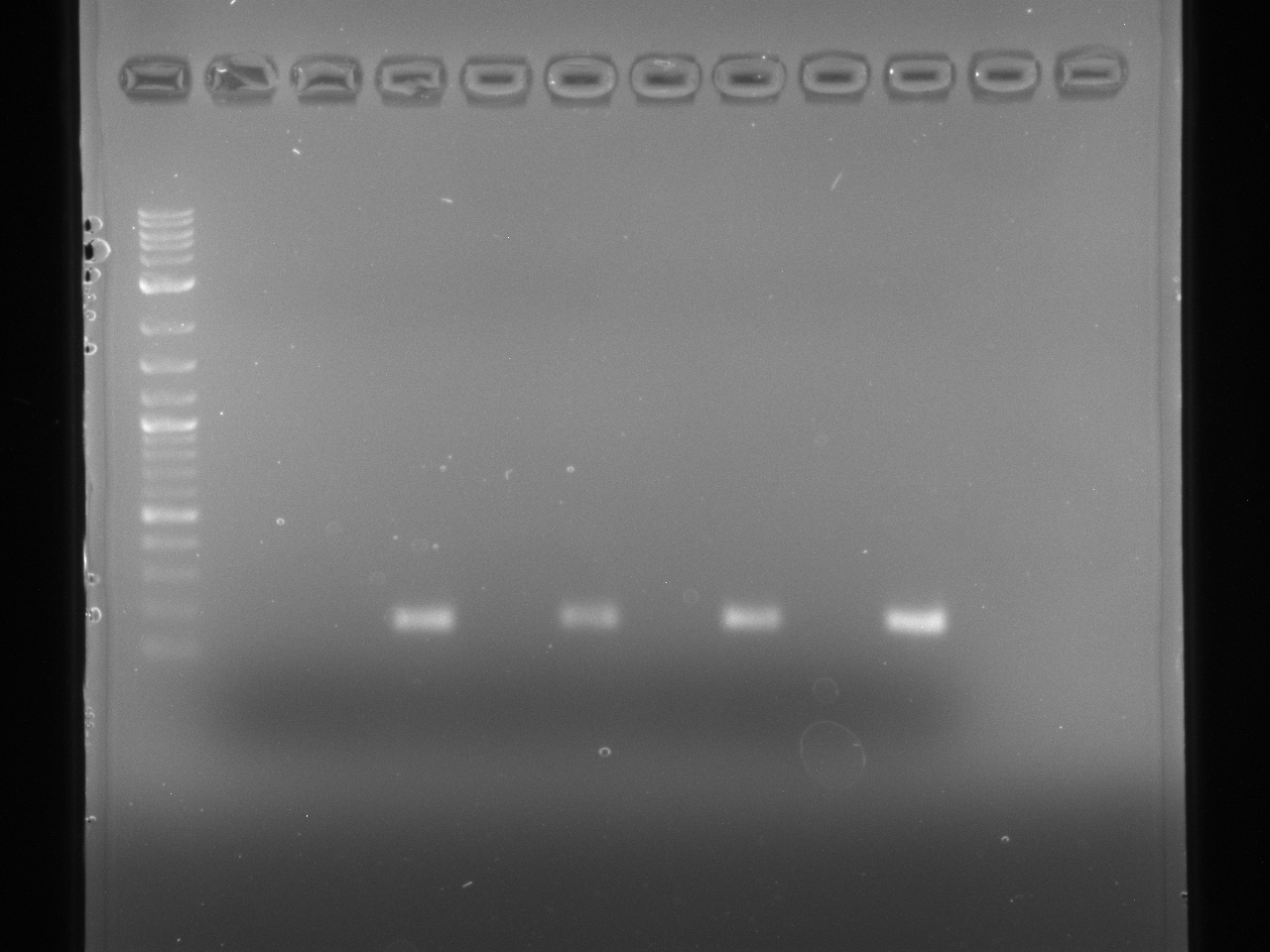

Supplement: Figure 3—source data 2. [file elife-74334-fig3-data2.zip › Figure_3_raw_gels_unedited/2020-11-03_09-07-36_AmOARbeta2.tif]

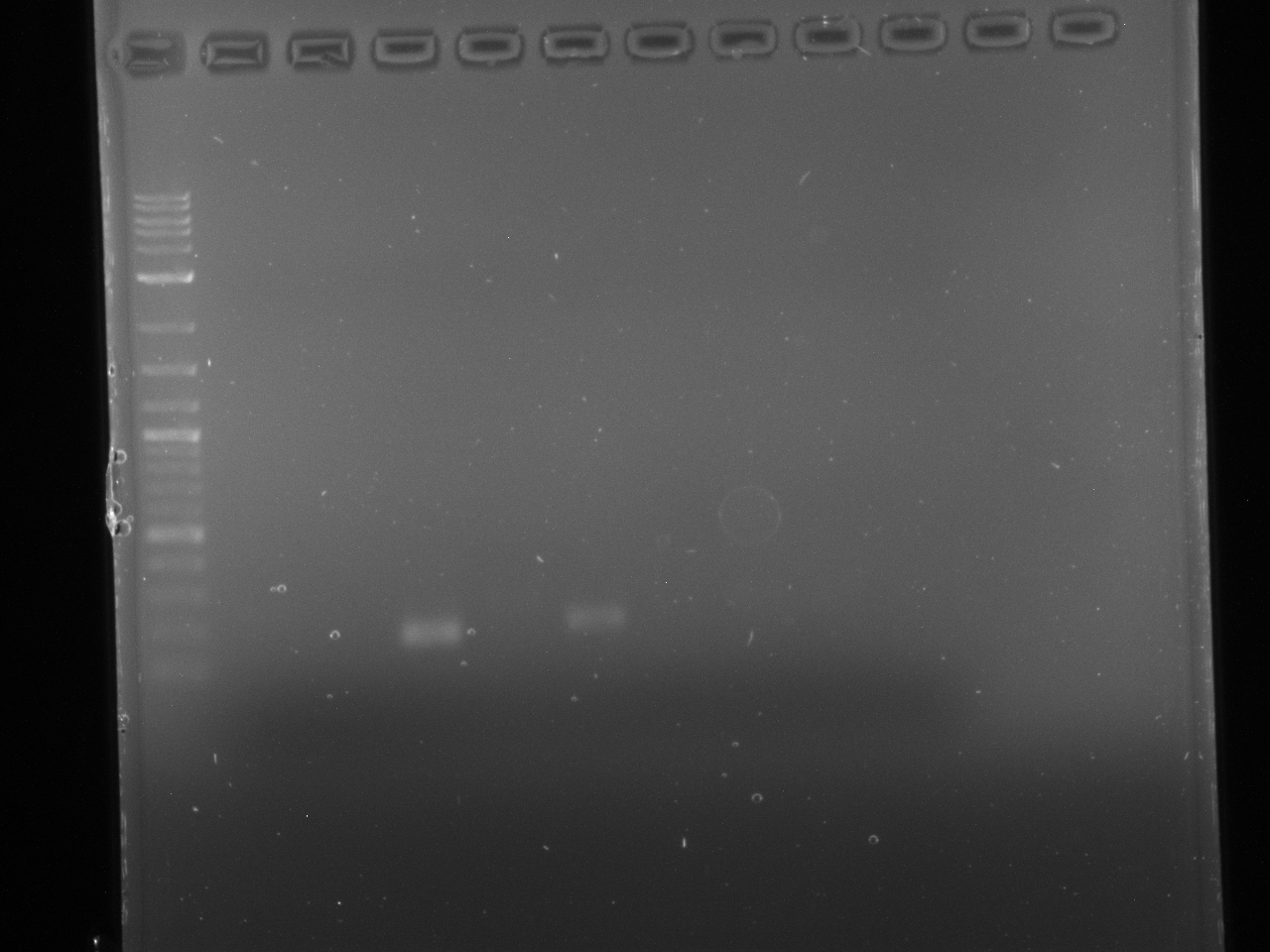

Supplement: Figure 3—source data 2. [file elife-74334-fig3-data2.zip › Figure_3_raw_gels_unedited/2020-11-03_10-18-19_AmTAR1.tif]

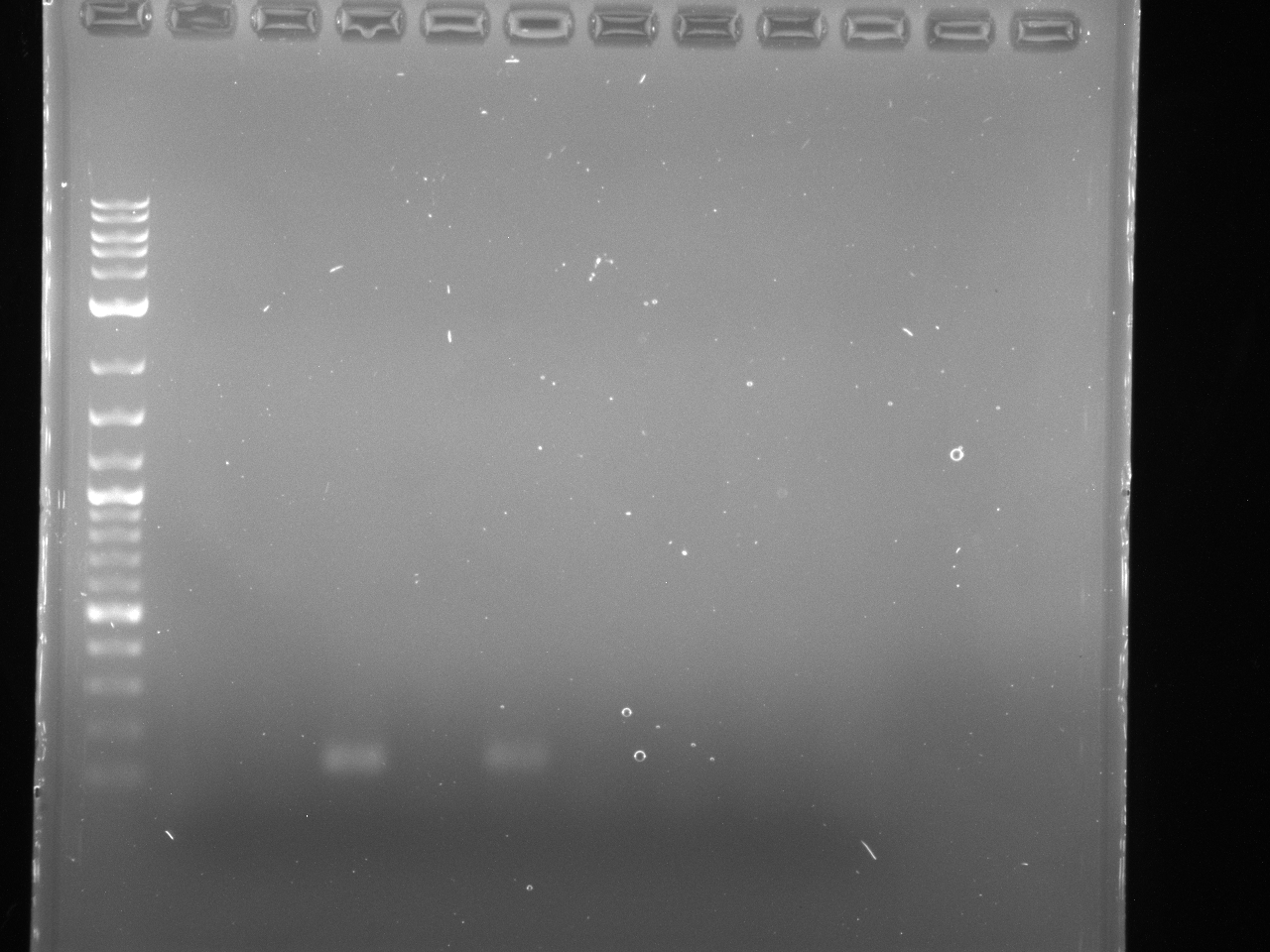

Supplement: Figure 3—source data 2. [file elife-74334-fig3-data2.zip › Figure_3_raw_gels_unedited/2020-11-02_12-45-58_AmTAR2.tif]

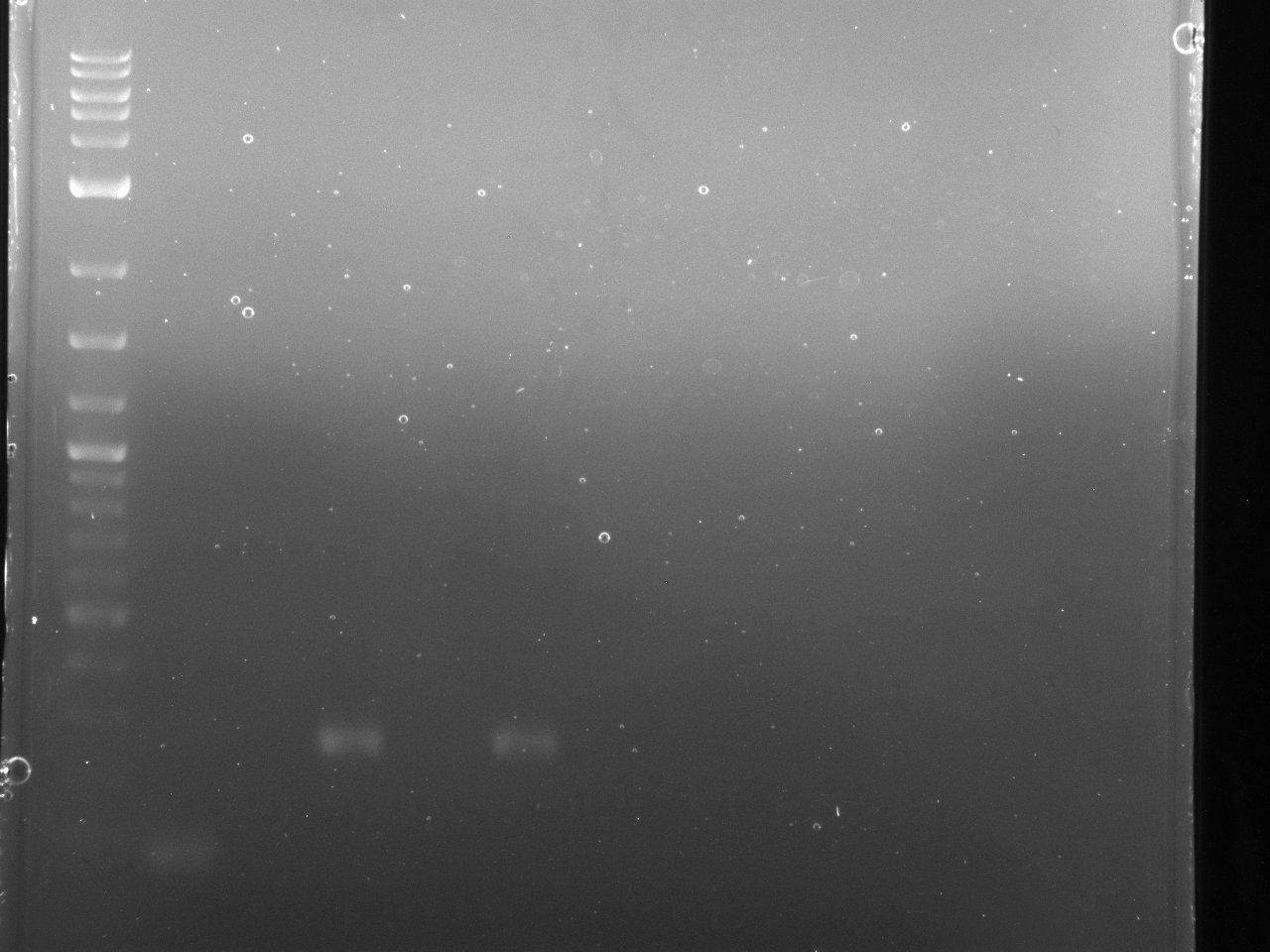

Supplement: Figure 3—source data 2. [file elife-74334-fig3-data2.zip › Figure_3_raw_gels_unedited/2020-11-05_10-52-27_AmOARalpha2.tif]
